# Efficacy of Sustained Acoustic Medicine as an Add-on to Traditional Therapy in Treating Sport-related Injuries : Case Reports

**Published:** 2020-09-21

**Authors:** David O Draper, Aaron Wells, Kevin Wilk

**Affiliations:** 1 Brigham Young University, USA; 2 Champion Sports Medicine, USA

**Keywords:** Low-Intensity Ultrasound, Continuous Ultrasound, Long-Duration Ultrasound, Athletic Training, Clinical Trial, Musculoskeletal Injury, Musculoskeletal Healing, Acute Pain, Chronic Pain

## Abstract

**Context:**

Musculoskeletal injuries are prevalent in sports, and the application of Sustain Acoustic Medicine (SAM) as a home-use add-on therapy to reduce pain and to increase the probability of athletes returning to sports was evaluated in a case series.

**Objectives:**

To examine the improvements in pain and return to function of athletes using SAM in conjunction with traditional therapies after sustaining sports-related musculoskeletal injuries.

**Introduction:**

Traditional treatments such as rest, physical therapy, manual therapy, a combination of rest, ice compression, and elevation (RICE) are standard of care for musculoskeletal injuries and do not provide adequate accelerated healing to return athletes to activity. SAM is an FDA-approved bio-regenerative technology, which can provide mechanotransductive and thermal stimuli to accelerate tissue healing and reduction in pain daily. Interventions: A case series of 18 athletes who showed little or no improvement with traditional therapies where prescribed SAM treatment as an add-on daily home-use intervention. The study included athletes with sports musculoskeletal injuries, including the arm/shoulder, upper leg/glutes/hips, knees, back, and foot/ankle. Clinical outcomes were recorded along with the ability of athletes’ ability to go back to sports, and satisfaction and usability measures of the home treatment. Results: All athletes were satisfied with the usability and comfort of the therapy and 93%reported the therapy was sufficiently discrete. Clinical outcomes indicate all athletes showed an average pain decrease of 3.33±0.82 (p≤0.05) numerical rating scales (NRS), improvement in function, and quality of life. 87% of the athletes documented an improvement in function, and 55% were able to return to sports after conservative intervention failed. Conclusion: The results of this study indicate that SAM improves athletes’ clinical outcomes. Over 50% of athletes were able to return to sports and resume normal daily function after conservative intervention had failed with addition of daily SAM treatment.

## Introduction

Musculoskeletal injuries are the most common injuries in recreational, amateur, and professional sports [1–4]. Approximately 64% of injuries in recreational sports are musculoskeletal related injuries [5]. Approximately 2 million injuries, 500,000 doctor visits, and 30,000 hepatization in high school students are associated with sports injuries [5,6]. The National Institute of Arthritis and Musculoskeletal and Skin Diseases has reported that 2.6 million children under 19 years old get treated for musculoskeletal injuries in emergency departments [7]. Hootman et al. reported that more than 50% of sports injuries in college students are in lower extremities with additional injuries reported in upper extremities and back/neck [8]. Historical data of American college football players show an increased number of upper shoulder and arm surgeries. Professional sports data is relatively discreet. Ekstrand et al. has reported that average soccer players sustained 2 injuries per season, leading to missing 37 days in a season [1,9]. Furthermore, 92% of all muscle injuries are associated with lower limb: hamstring (37%), adductors (23%), quadriceps (19%), and calf muscles (13%)[10]. A recent study by Humprey et al. reported professional tennis players to sustain most of their injuries at muscles (24%), tendon (23.4%), and soft tissue bruising (6.5%)[3]. Detailed studies have shown that the overuse of musculoskeletal anatomy further adds to injuries and probabilities of the future of reinjuries [1].

The musculoskeletal healing process involves activation of the cascade of cellular and molecular pathways [11, 12]. The initial response to the injury is the activation of inflammatory cytokines. Acute inflammation is an essential part of the healing process, but chronic inflammation leads to the delayed healing process as well as further damage to the tissue; thus, it is necessary to remove inflammatory cytokines and initiate the regeneration of new tissue. Currently, to restrain the level of inflammation and enhance tissue healing, athletic trainers most commonly use rest, ice compression, and elevation (RICE) method. RICE reduces the inflow of the inflammatory factors, along with reducing the blood flow to the injury site [13,14]. RICE has limited efficacy in reducing inflammation and improving the rate of tissue regeneration over the healing process. Still, there is a need for add-on therapies to expedite the healing process and tissue regeneration [15–18].

Post-injury, the most important concern for an athlete is when to “return to play”? [19] This is a hard question to answer considering the multifactorial factors such as the site of injury, damage sustained, athletes’ age, and the number of revised injuries along with other potential health concerns [19,20]. Doctors and athletic trainers are advised to have a conservative approach, but at the same time, it important to minimize the athlete’s off-field time. Thus, it is important to come with more advance, innovative, and non-invasive therapies to accelerate tissue healing.

Ultrasound therapy provides a mechanotransducive localized stimulus to the injury site resulting in increased blood flow, oxygenation, exchange of nutrition, and accelerated tissue healing resulting in inhibition of inflammation [21–25]. Multiple studies have shown tissue regenerative properties of ultrasound. Ultrasound increases the rate of bone healing by decreasing initial infiltration of cytokines increasing, angiogenesis, chondrogenesis, and bone remodeling [22,23,26,27]. Studies have concluded the application of ultrasound increases the rate of tendon-bone interface healing in preclinical models [28–31]. Walsh et al. has shown similar trends in anterior cruciate ligament reconstruction [31].

Ultrasound is a non-invasive therapy with significant data supporting its regenerative efficacy in preclinical studies [21,28,32–34]. The effectiveness of ultrasound is dependent on multiple factors such as duty cycle, intensity, duration, frequency, and energy [21,35–37]. While ultrasound therapy has been around for a long time, its transformation to clinical practice has been limited due to the optimization of ultrasound parameters. Low-intensity continuous ultrasound (LICUS) has shown the potential to accelerate the tissue regeneration process significantly. Sustained Acoustic Medicine (SAM) uses LICUS to expedite the healing process and have shown encouraging results in clinical studies [28,38–43]. SAM® has been approved for pain management by Food and Drug Management (FDA). Langer et al., in a clinical study (n=30) has shown that after use application of SAM (3MHz, 0.132W/cm2, 18720 Joules) for 4 hours reduced pain by 15% compared to 7% in the placebo group in Trapezium muscle spasm. They also showed 52% (p<0.05) improvement in the visual analog score (VAS) in rotator cuff pain, 40% (p<0.03) pain reduction in osteoarthrosis patients (N=47), and 4.28 point VAS (p<0.001) decrease in tendon pain relief and recovery (N=25) after 6 weeks of treatment [41,42]. Lewis et al. has reported an up to 1point increase in Global Rating of Change (GROC) score and a 25% reduction in VAS reduction after 10 days of SAM treatment in chronic myofascial pain [43]. The objective of this study is to evaluate the effects of SAM in sports-related injuries as an adjuvant therapy to athletes when conservative treatment fails.

## Materials and Methods

### Participants

**Figure 1:**
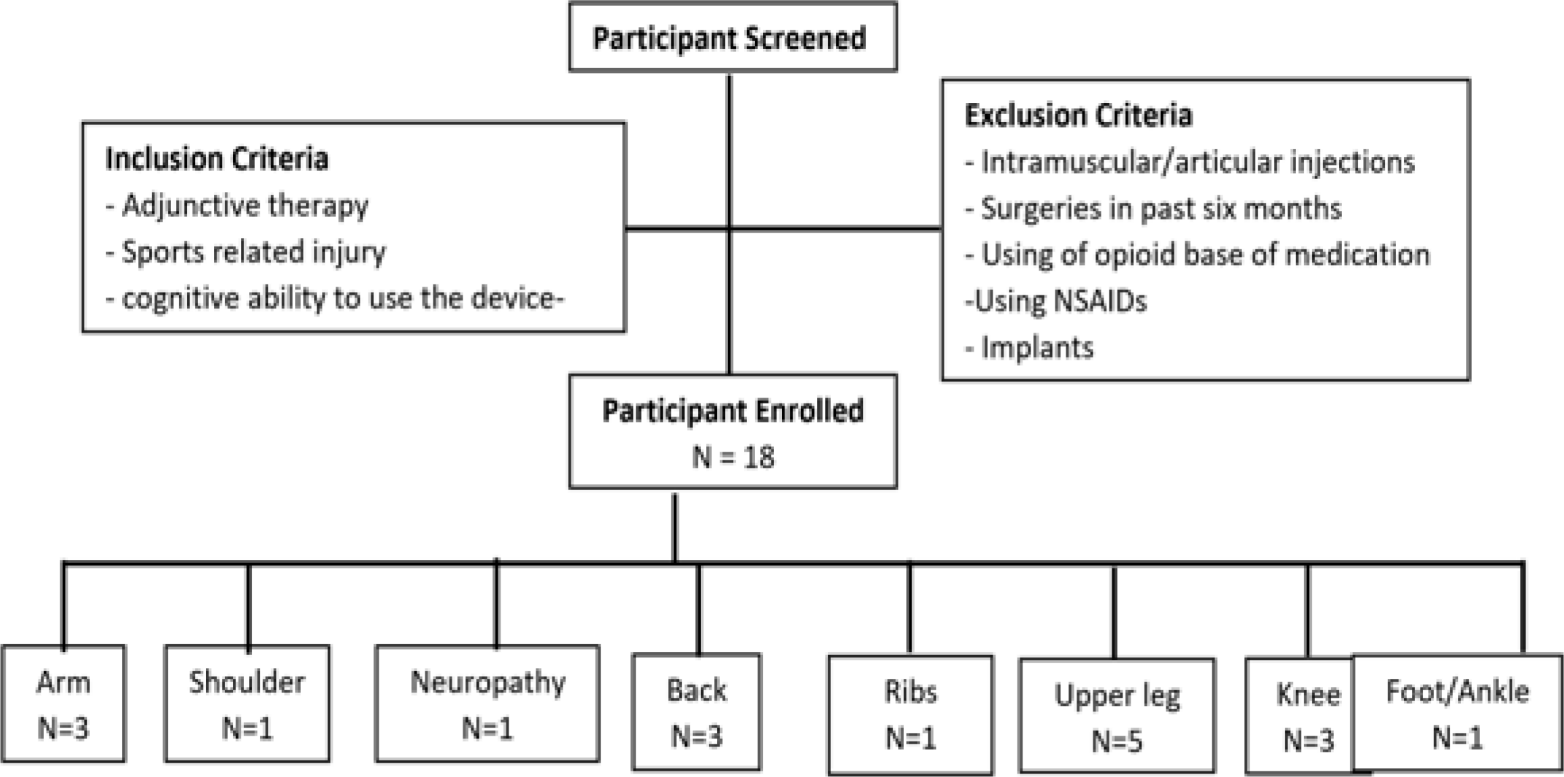
Participant criteria and sites of musculoskeletal injury treated.

Participants (N=18) included amateur and professional athletes from different sports who did not respond to traditional musculoskeletal therapies with an average age of 30±13.31 (13 Males, 5 Female) Table 1. Participate were screened before the study and excluded from the study if they 1) had surgeries, 2) used opioid-based medication, 3) any implants, 4) Intramuscular or articular steroid injections, and 5) using systemic/oral non-steroidal anti-inflammatory drugs (NSAIDs). The participants were included if they 1) using adjunctive therapy, 2) sports-related injury, and 3) cognitively able to follow the instruction to use the medical device. The participants included athletes with arm injuries (n=3), shoulder (n=1), neuropathy (n=1), back (n=3), ribs (n=1) upper leg (n=5), knee (n=3), and foo/ankle (n=1) Figure 1. The participants signed the consent form, and observations were conducted per declaration for Helsinki [44] (Table 1) (Figure 1).

### SAM Device

**Figure 2:**
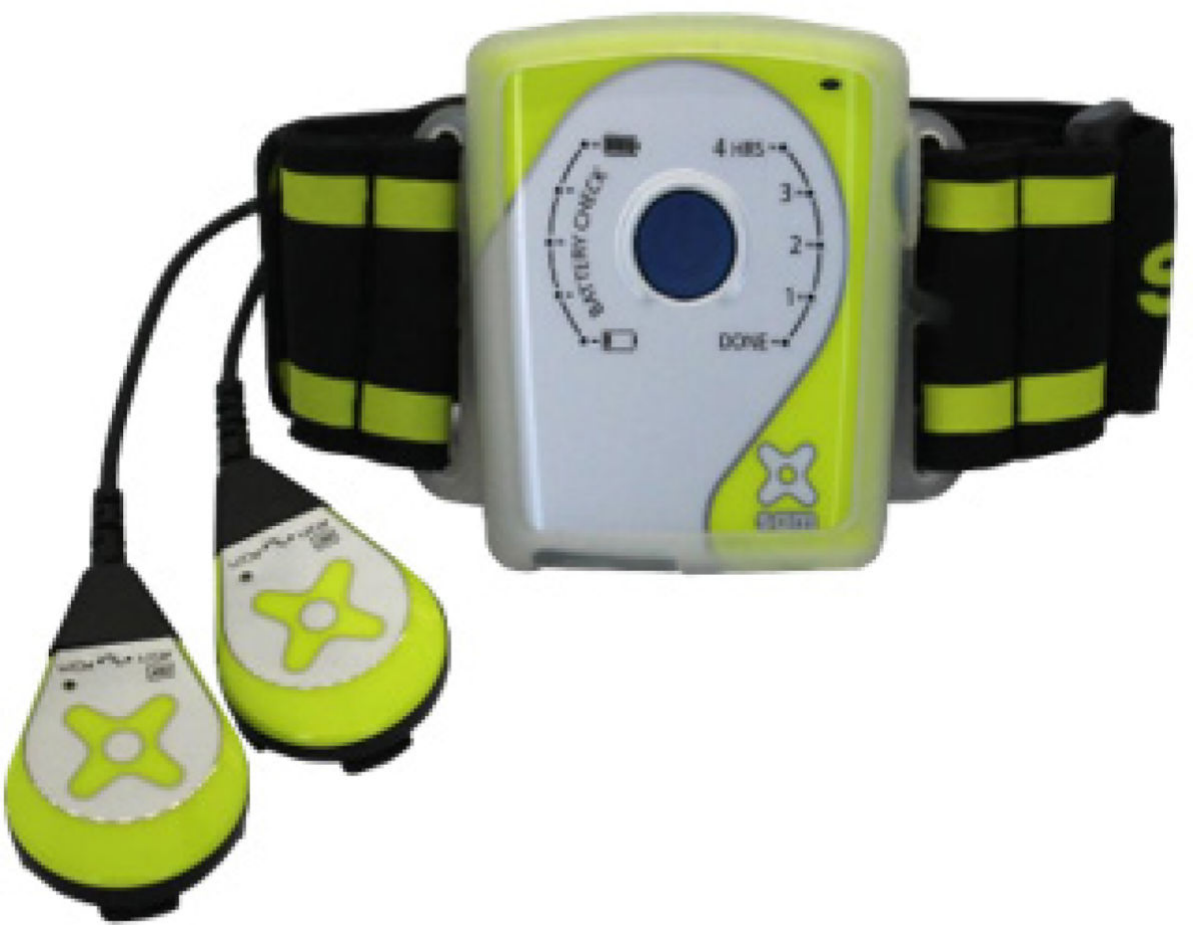
The SAM® device Two transducers (Crystals) and one power controller in an arm strap. Two transducers deliver ultrasound at 3MHz, 0.132W/cm2, 1.3W, each delivering total of 18720 Joules over 4 hours of treatment.

**Figure 3:**
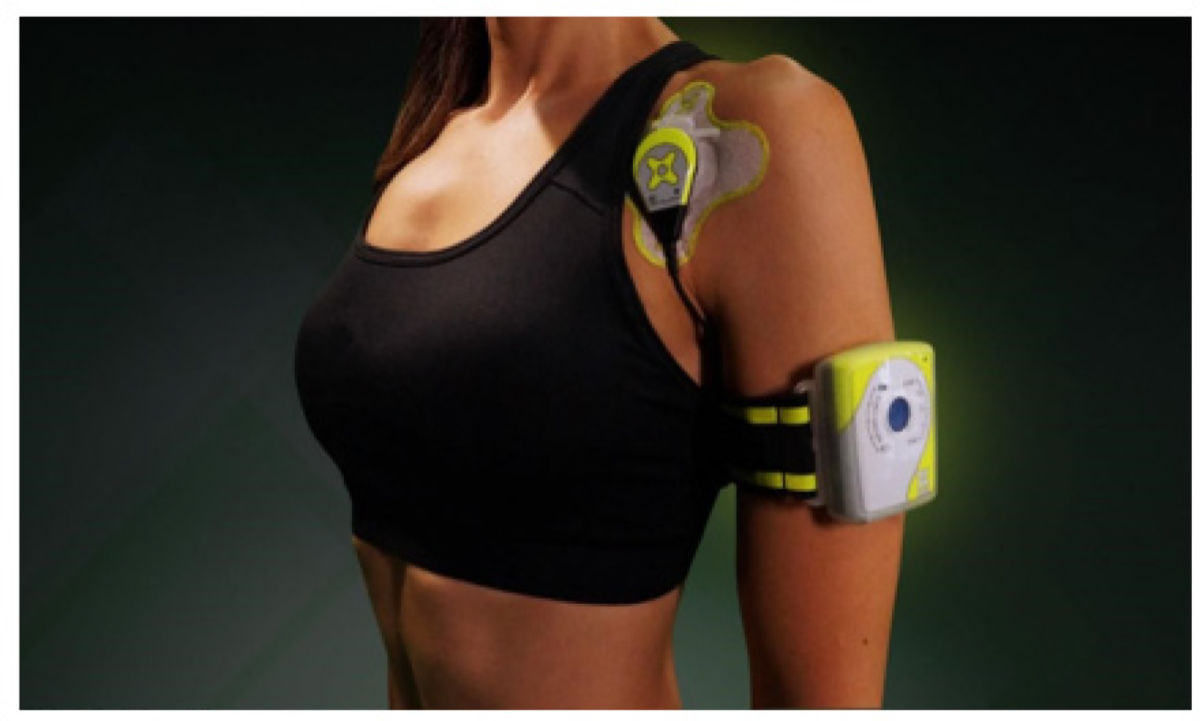
Application of SAM® to the shoulder.

The LICUS was delivered using an FDA approved SAM® (ZetrOZ Systems LLC, Trumbull, CT) (Figure 2). The device consists of two transducers that deliver ultrasound at 3MHz, 0.132W/cm2, 1.3W, each delivering total of 18720 Joules over 4 hours of treatment. Athletes were trained to use how to use the device properly with an ultrasound gel adhesive patch by trained medical staff (Figure 2,3).

## Result

### Device use and utility

17 out of 18 athletes found SAM® user friendly, one athlete reported olecranon bursitis. Two athletes reported minor skin irritation due to the adhesives used (Table 2).

### Clinical outcomes

The objective of this report was to examine the clinical change in pain associated with injury after treatment with SAM® and return to regular functional activity or sports after the failure of conservative therapy (Table 3). All athletes (100%) stated they had a benefit from using the device and had an improvement in pain with the average reported a decrease of 3.33±0.82 (p ≤0.05) numerical rate scale (NRS) on the scale of 1 – 10 for 6 patients who reported pain score. All other patients reported the effectiveness of SAM® treatment. Sixteen (16) out of 18 athletes (89%) indicated an improvement in range of motion, and 10 out of 18 (55%) reported to return to sporting activities after approximately 2.25 weeks of intervention. Case Results by Injury Site

### Arm

#### Elbow1

Two patients were treated with SAM® for elbow injuries. The first athlete was a 38-year old male athlete with chronic left epicondylitis with pain worsened during movement and gripping. Traditional physical therapy and stretching were unsuccessful in rehabilitation. The addition of SAM® for 4 hours per day for 1 week resulted in significant relief of pain and resulting in a return to sporting activities.

#### Elbow2

The second athlete was a 32-year old male with olecranon (elbow) bursitis associated with physical exercise lasting 2 weeks. Minimal change in symptoms were reported with ice and rest. The addition of SAM® to his treatment regimen for 4 hours /day, for 2 weeks resulted in a reduction in pain severity. This athlete had an overall improvement in symptoms that allowed him to “perform activities of daily living and work activities with reduced pain.” Radius. A 17-year old female softball pitcher with an acute injury was treated with SAM®; she had a contusion of her left radius (non-throwing arm) due to direct impact, resulting in sharp, localized pain that was further aggravated by continuing to pitch. The icing alone was inadequate to relieve pain. She was treated with soft tissue mobilization and SAM® (4 hours daily for 1 week), leading to an overall improvement in pain and enabling her to return to softball.

### Shoulder

A 32-year old male professional baseball pitcher with chronic (2 years) supraspinatus tendinosis and impingement syndrome were treated with heat, ice, rest, stretching, electrical stimulation, and manual therapy for 2 years. The addition of SAM® ( 4 hours/day) for 3 days per week for 1 week, along with muscle therapy, increased range-of-motion, and reduced pain (Figure 2).

### Neuropathy

A 35-year old male reported sharp pain with numbness/tingling in his left arm that radiated to his hand. The neuropathic pain was present in rest and at gripping motion and daily function. Prior treatment with typical physical therapy and stretching, as well as therapeutic ultrasound, were unsuccessful at relieving pain. This patient was treated with SAM® for 4 hours/day for 2 weeks, along with manual therapy, therapeutic exercise, resulting in a significant decrease in pain. He displayed an increased range of motion, decreased tissue density, and increased strength and gripping while a reduction in tingling and numbness.

### Back

#### Herniated Disc 1

A 20-year old female amateur rower with a lumbar disc herniation, reported sharp and radiating pain, with numbness and tingling lasting 2–3 weeks at the time. This athlete was treated with SAM® for 4 hours/day for 2 weeks, in combination with manual therapy, and therapeutic exercise. The athlete reported relief of pain and an increased range of motion. She was able to attain “pain-free daily function, and to resume running and working out.”

#### Herniated Disc 2

A 54-year old male professional golfer with 46 years in the sport who had a lumbar disc herniation. He had temporary pain relief with manual therapy, ice, and muscle activation sufficient to participate in sport, but symptoms inevitably returned within approximately 2 weeks. The SAM® was added (4 hours/day, 5 days per week) into his treatment regimen, along with manual therapy and muscle activation for 2 weeks. The athlete reported, SAM® as a valuable treatment alternative for times when muscle activation therapy was not available.

#### Herniated Disc 3

A 45-year old male amateur basketball player with chronic low back pain that worsened with play (during and after basketball). Manual therapy combined with using SAM® 4 hours/day, 5 days/week for 2 weeks resulted in marked pain relief; specifically, he improved from having constant pain during exercise to having no pain during and after playing basketball with combined treatment. As a result of the decrease in pain, the athlete was able to have longer endurance on the court leading to improved overall function and improved active field time.

### Ribs

An 18-year old male amateur hockey forward was treated for a bruise to the 12th rib. No pain was reported at rest, but sharp pain was reported at the movement. X-rays were negative for fracture. Initial therapy with manual therapy, heat, and nutritional changes yielded minor positive changes. However, when SAM® was added (4 hours/day, 4 days over 1 week), the combined therapy changed the quality of his pain from “sharp” to “dull” and activity from moderate to mild.

### Upper Leg

Piriformis. 16-year old male soccer goalkeeper with chronic (>1 year) piriformis syndrome affected his right hip. Traditional physical therapy, including stretching and use of therapeutic ultrasound, was unsuccessful at alleviating his symptoms. The combination of manual therapy, therapeutic exercises, education, in conjunction with the SAM®, used for 4 hours/day for 2 weeks, resulting in pain relief, reduction in strained tissue, and a return to functional goalie activities.

Hip Flexor/Quadriceps Strain. A 30-year old male professional baseball player (shortstop) with 20 years of experience in the sport. He had a hip flexor/quadriceps strain with sharp pain on extension. Conservative therapy with ice and heat yielded negligible improvement in symptoms. After 2 weeks of symptoms, the addition of SAM® therapy for 4 hours/day, 5 days over 1 week, resulted in a reduction of pain and increased range-of-motion with extension.

#### Hamstring 1

A 15-year old female amateur soccer midfielder with more than 5 years in the sport. She suffered right hamstring strain with chronic symptoms (>1 year) resulting from an apparent biomechanical imbalance. She reported sharp pain in the hamstring, particularly with running. Conservative physical therapy had been unsuccessful. Manual therapy, therapeutic exercises, and education were combined with daily SAM treatment (4 hours/day) for 1 week. The athlete then continued to use SAM® sporadically for over 2 months. The combined treatment protocol led to significant pain relief, allowing her to increase her speed and to resume soccer activities.

#### Hamstring 2

A 32-year old male professional baseball player (2nd baseman) with 22 years in the sport. He was treated for a hamstring tear (biceps femoris) with minimal benefit perceived from strength, range-ofmotion, and massage therapy. A 3-week treatment with SAM® (4 hours/day, 5 days/week) together with manual therapy and muscle activation resulted in pain relief and contusion reduction and a change in the quality of the residual pain from “sharp” to “dull.”. This athlete reported that he “could feel an immediate impact of treatment” during and after the treatment sessions by the heating of the tissue and the increased range of motion (Figure 3).

##### Tendinopathy

A 60-year old female with chronic gluteus medius tendinopathy associated with physical exercise. The pain was worsened by loading of the leg going up and downstairs, and by single-leg exercises. She was treated with manual therapy, muscle activation, ice, and SAM® (4 hours/day, 6 days in 1 week). She reported mild pain relief with treatment with a reduction in pain severity.

### Knee

Patellar Tendinosis. A 37-year old male amateur marathoner, who was new to the sport. He developed patellar tendonitis of his right knee and has had symptoms lasting 2–3 months. The pain was worsened by running and weight-bearing. Conservative treatment with RICE showed little effects. The addition of SAM® to RICE for 4 hours/day resulted in pain relief and functional improvement, enabling him to run and compete in the Boston Marathon.

#### Anterior Cruciate ligament tear

A 17-year old female basketball shooting guard with an anterior cruciate ligament (ACL) tear of her right knee underwent knee surgery. After surgery, RICE, and an ACL rehabilitation program, she did not have any change in the tendon symptoms. SAM® (4 hours/day) was then added to her treatment regimen for 2 weeks resulting in an improvement in pain and swelling, allowing her to continue to progress through the ACL rehabilitation program.

#### Osteochondral Fracture

A 24-year old male professional football player (right tackle) with a right medial osteochondral fracture. He was treated with microfracture surgery, RICE, soft tissue mobilization, and neuromuscular re-education yielding limited effectiveness on reduction of pain. Addition of SAM (4 hours/day for 4 weeks) the athlete reported reduced pain and tightness in the injured knee, allowing him to progress through rehabilitation effectively.

### Foot/Ankle

A 19-year-old male amateur lacrosse player was treated with SAM® for bilateral distal posterior tibial tendinopathy. The athlete has been dealing with overuse injury for approximately 12 weeks and had not improved with conservative therapy (RICE). The addition of SAM® for 4-hour daily treatments for 4 weeks applied to the ankles, in combination with RICE, soft tissue mobilization, and therapeutic exercise. The athlete reported improvements in pain and tightness with treatment. He experienced a reduction in pain and was able to return to lacrosse.

## Discussion

The current study describes a case series of 18 athletes treated with SAM® as adjunct therapy at a single sports medicine rehabilitation clinic. SAM® treatment resulted in reduced pain and improved function across numerous muscles, ligament, and tendon conditions. Most of the athletes examined were able to return to normal activity and function, including sports, during their treatment period. These athletes previously underwent surgeries or were being considered for surgery.

These studies used SAM® (ZetrOZ System LLC) device with two transduces to deliver SAM® at 3MHz, at an intensity of 0.132W/cm2 per transducer. Delivering 18720 Joules of energy over 4 hours of treatment. Multiple studies have shown the efficacy of SAM® device as an efficient technology to provide consistent SAM® over 4 hours [28,38–41,43,45]. Lewis et al. have demonstrated a constant acoustic signal and its effect on chronic myofascial pain, with a 25% reduction in the pain after 10 days of treatment and a 30 % decrease in rotator cuff tendinopathy after 12 treatments [43,46]. Similarly, best et al. has shown 3.94 (n=20, p<0.002) point decrease point reduction in elbow tendionopathathy (n=20) and 2.83 kg increase grip strength (p=0.02) after 6 weeks of treatment of SAM®[47]. SAM has also been shown to be effective for osteoarthritis.

Langer et al. showed a statistically significant 20% mobility in arthritic patients after 4 weeks of patients with SAM®[41]. In a clinical study, Draper et al. determined the efficacy of SAM® with 90 arthritis patients. After 6 weeks of treatment, patients treated with active SAM® show 1.96 points (p<0.0001) decrease in the Numerical Rating Scale (NRS) of pain relative to 0.85 points in the placebo group. The Western Ontario McMaster Osteoarthritis Index (WOMAC) score also improved by 505 points in the active group relative to 311 points in the placebo group (p=0.02) [38].

The data for the effectiveness of ultrasound for inhibition of inflammation through inhibiting cytokines, recruiting stem cells to injury sites, increasing the rate of tissue regeneration through matrix formation via collagen alignment, and other matrix protein has been shown for decades in preclinical studies [48]. Still, the translation of ultrasound to clinical application has lagged [48]. One of the factors causing lagging of the ultrasound technology from preclinical to clinical application is the optimization of its parameters. The current studies with SAM® provide us with encouraging data showing the effectiveness of ultrasound as in the clinical setting and its translation from preclinical to real-life clinical settings [28,38,40,43,46,49,50].

The clinical data in this study confirms the effectiveness of the application of therapeutic ultrasound in reducing pain as adjunct therapy or standalone therapy. Muffic et al have shown the effectiveness of different intensities of ultrasound in pain associated to degenerative musculoskeletal diseases [51]. Although, a recent study by Noor et al. failed to draw any conclusive recommendation about application ultrasound in treatment pain management due to heterogeneity of conditions such as duration, intensity, frequency, and mode of application [52]. Multiple meta-analyses show that low-intensity therapeutic ultrasound is beneficial for chronic pain [35, 53–55].

SAM® is an optimized low-intensity continuous therapy that accelerates the natural process of healing by inhibiting inflammation, increase the rate of tissue regeneration, angiogenesis, and nutrient exchange. The application of LICUS through SAM®, the portable device makes it easy for athletes to use it as an add-on device to active recovery regime and accelerate the rate of recovery and probability of returning to the sport.

## Conclusion/Summary

Sport-related musculoskeletal injuries are highly prevalent and require novel approaches to accelerate healing. Traditional therapies may be insufficient for work-related injuries, causing athletes to suffer through pain and a long time to return to sporting activities. RICE, along with other therapies, has been used to treat musculoskeletal injuries. This study describes a case series of injured athletes receiving SAM® as an add-on therapy to traditional treatments to accelerate the healing process and decreases the pain and time to return to sporting activities as well as the overall quality of life.

## Figures and Tables

**Table 1:** Athlete cohort and demographics.

Location	Diagnosis	Age	Gender	Sport
Arm	Left epicondylosis	38	M	Gym/exercise
Arm	Olecronon bursitis	32	M	Gym/exercise
Arm	Radial contusion	17	F	Softball
Shoulder	Chronic supraspinatus tendinosis, impingement	32	M	Baseball
Neuropathy	Left-arm neuropathy	35	M	Unknown
Back	Lumbar disc herniation	20	F	Crew
Back	Lumbar disc herniation	54	M	Golf
Back	Low back pain	45	M	Basketball
Ribs	12^th^ rib bone bruise	18	M	Hockey
Upper Leg	Right hip piriformis syndrome	16	M	Soccer
Upper Leg	Hip flexor/quadriceps strain	30	M	Baseball
Upper Leg	Right hamstring strain	15	F	Soccer
Upper Leg	Hamstring tear	32	M	Baseball
Upper Leg	Gluteus medius tendinopathy	60	F	Gym/exercise
Knee	Patellar tendinosis	37	M	Running
Knee	Right knee; ACL tear	17	F	Basketball
Knee	Osteochondral fracture	24	M	Football
Foot/Ankle	Distal posterior tibial tendinopathy	19	M	Lacrosse

**Table 2:** Device Use.

Device Use	Yes	No
Was the device easy to use? Would you describe sam as “userfriendly”?	18	0
Is the device sufficiently discrete?	17	1
Is the device comfortable to wear while going about daily activities?	18	0
Did the patient experience any skin sensitivity?	2	16

**Table 3:** Clinical Outcomes demonstrating changes in pain, functionality and return to sports post LIPUS addon treatment.

Location	Diagnosis	Treatments Per Week	Pre-Sam Treatment	Effects	Additive Treatment + Sam	Treatment Timeline (Weeks)	Pain Reduction	Increased ROM/Function	Returned To Work/Sports
Arm	Left epicondylosis	7	Ice. Rest.	Minimal Change	Ice. Rest.	1	Yes	Yes	Yes
Arm	Olecronon bursitis	7	Typical PT, stretching	No Change	manual therapy, therapy education	2	Yes	Yes	No
Arm	Radial contusion	7	Ice	No change	manual therapy, therapy education	1	Yes	Yes	Yes
Shoulder	Chronic supraspinatus tendinosis, impingement	7	Heat, Ice, rest, stretching, electrical stimulation, manual therapy	No change	Muscle therapy, muscle activation	2	Yes	Yes	Yes
Neuropathy	Left-arm neuropathy	7	Typical PT, stretching	No Change	manual therapy, therapy education	2	Yes	Yes	Yes
Back	Lumbar disc herniation	5	Typical PT, stretching	No Change	manual therapy, therapy education	1	Yes	Yes	No
Back	Lumbar disc herniation	6	Strength, ROM, Massage	No Change	Manual Therapy, Muscle Activation	1	Yes	Not Significant	No
Back	Low back pain	5	Typical PT, stretching	No Change	manual therapy, therapy education	2	Yes	Not Significant	No
Ribs	12th rib bone bruise	4	Manual therapy, heat, nutritional changes	Minimal Change	Physical therapy	1	Yes	Yes	Yes
Upper Leg	Right hip piriformis syndrome	5	Ice, Heat	No Change	Muscle therapy, heat	3	Yes	Yes	No
Upper Leg	Hip flexor/quadriceps strain	7	None	None	manual therapy, therapy education	2	Yes	Yes	Yes
Upper Leg	Gluteus medius tendinopathy	7	Surgery. RICE. ACL rehab	No Change	Ice. Soft tissue mobilization. ACL rehab	1	Yes	Yes	Yes
Upper Leg	Right hamstring strain	7	Manual therapy, Muscle activation, Ice	Minimal Change	Manual Therapy, Muscle activation, Ice	8	Yes	Yes	Yes
Upper Leg	Hamstring tear	3	Rest, ice, compression	No change	manual therapy, therapy education	1	Yes	Yes	No
Knee	Patellar tendinosis	7	Manual therapy, ice, muscle activation	Minimal Change	Manual therapy, muscle activation	4	Yes	Yes	Yes
Knee	Right knee; ACL tear	7	Manual therapy, Heat pack, nutrition changes	Minimal Change	Manual therapy	2	Yes	Yes	Yes
Knee	Osteochondral fracture	7	MicroFracture Surgery/RICE/Soft Tissue mobilization/Neuromuscular Reeducation	Minimal Change	Ice. Soft tissue mobilization. ACL rehab	7	Yes	Yes	Yes
Foot/Ankle	Distal posterior tibial tendinopathy	4	Ice. Rest.	No Change	Ice. Soft tissue mobilization. Therapeutic Exercise	-	Yes	Yes	No

## Abbreviations

RICE: Rest, Ice, compression, and Elevation
NSAIDs: Non-Steroidal Anti-Inflammatory Drugs
LICUS: Low-intensity continuous ultrasound
SAM: Sustained Acoustic Medicine
ACL: Anterior Cruciate Ligament
NRS: Numerical Rating Scale
WOMAC: Western Ontario McMaster Osteoarthritis Index
